# Biosynthesis and Regulation of Wheat Amylose and Amylopectin from Proteomic and Phosphoproteomic Characterization of Granule-binding Proteins

**DOI:** 10.1038/srep33111

**Published:** 2016-09-08

**Authors:** Guan-Xing Chen, Jian-Wen Zhou, Yan-Lin Liu, Xiao-Bing Lu, Cai-Xia Han, Wen-Ying Zhang, Yan-Hao Xu, Yue-Ming Yan

**Affiliations:** 1 College of Life Science, Capital Normal University, 100048 Beijing, China; 2 Hubei Collaborative Innovation Center for Grain Industry, Yangtze University, 434025 Jingzhou, China

## Abstract

Waxy starch has an important influence on the qualities of breads. Generally, grain weight and yield in waxy wheat (*Triticum aestivum* L.) are significantly lower than in bread wheat. In this study, we performed the first proteomic and phosphoproteomic analyses of starch granule-binding proteins by comparing the waxy wheat cultivar Shannong 119 and the bread wheat cultivar Nongda 5181. These results indicate that reduced amylose content does not affect amylopectin synthesis, but it causes significant reduction of total starch biosynthesis, grain size, weight and grain yield. Two-dimensional differential in-gel electrophoresis identified 40 differentially expressed protein (DEP) spots in waxy and non-waxy wheats, which belonged mainly to starch synthase (SS) I, SS IIa and granule-bound SS I. Most DEPs involved in amylopectin synthesis showed a similar expression pattern during grain development, suggesting relatively independent amylose and amylopectin synthesis pathways. Phosphoproteome analysis of starch granule-binding proteins, using TiO_2_ microcolumns and LC-MS/MS, showed that the total number of phosphoproteins and their phosphorylation levels in ND5181 were significantly higher than in SN119, but proteins controlling amylopectin synthesis had similar phosphorylation levels. Our results revealed the lack of amylose did not affect the expression and phosphorylation of the starch granule-binding proteins involved in amylopectin biosynthesis.

Wheat is one of the most important grain crops in the world, and the dry seeds have approximately 65–75% starch^1^. Flour starch of bread wheat usually consists of about 25% amylose and 75% amylopectin, whereas waxy starches have low amylose content (less than 3%) and very high amylopectin levels^2^. Waxy and non-waxy wheat can be easily distinguished by a staining method. Starch of non-waxy lines containing amylose forms blue-black complexes with iodine, while starch of waxy mutants without amylose stains red-brown^3^,^4^. Incorporation of waxy wheat flour in a bread formulation resulted in retention of moisture in breadcrumbs and a delay in bread staling, resulting in the extension of bread shelf life^5^,^6^,^7^. In addition, waxy wheat flour could influence the bread dough and qualities of breads, and waxy flour could be used for bread making to improve the nutritious quality of bread and exert beneficial effects on health^8^. Thus, the amylose content in wheat flour has been the focus of studies on flour quality, food products, and breeding^9^.

Amylopectin consists mainly of long chains of (1–4)-linked d-glucopyranosyl units with occasional branching (1–6) linkages, resulting in tandem linked clusters (each ~9–10 nm long), whereas amylose is a relatively linear molecule consisting of (1–4)-linked units of d-glucopyranosyl^10^. Amylopectins are synthesized via concerted reactions catalyzed by four enzymes, namely ADP-glucose pyrophosphorylase (AGPase), starch synthase (SS), starch-branching enzyme (SBE), and starch-debranching enzyme (DBE). Amylose synthesis is controlled by granule-bound starch synthase (GBSS I^11^) encoded by *Wx-A1*, *Wx-B1* and *Wx-D1* located on chromosomes 7AS, 4AL and 7DS, respectively^12^. The lack of *Wx-B1* has the greatest effect on the synthesis of amylose, followed by *Wx-D1* and *Wx-A1*^13^. There are eight combinations or types of null alleles of three *Wx* loci in wheat. The type with all three alleles has 20–25% amylose content. Types with one or two null alleles have 1.7–5.0% amylose content, and the type with all three null alleles has an amylose content of 0.6–0.7%^12^,^14^,^15^. Previous research in barley (*Hordeum vulgare* L.) indicated that GBSS I overexpression could increase the expression levels of SBE I, SBE IIa and SS I^16^. Efforts to generate high-amylose wheat varieties have focused on identifying alterations in a number of genes involved in the synthesis or branching of amylopectin. Null alleles of SS IIa in each of the A, B and D genomes were identified and combined to produce a wheat variety with a 10% increase in amylose content, from 25 to 35%. An RNAi construct targeting SBE IIb also increased the amylose content from 25 to 35%^17^. Similarly, in durum wheat, RNAi suppression targeting SBE IIa led to a 30 to 75% increase in amylose content^18^.

Protein phosphorylation, the most common *in vivo* post-translational modification, regulates and controls various biological processes such as transcription and translation, cellular signaling and communication, proliferation and differentiation. In eukaryotes, phosphorylation occurs mainly at serine (Ser), threonine (Thr), and tyrosine (Tyr) residues. The phosphoprotein detection of starch granule-binding proteins is accomplished primarily by three methods: Pro-Q Diamond staining, *in vitro* phosphorylation isotope labeling by γ-^32P^-ATP, and LC-MS/MS technology^19^,^20^,^21^,^22^,^23^,^24^. Grimaud *et al*.^22^ indicated that GBSS I could be stained with a phosphoprotein-specific dye in maize (*Zea mays* L.). Tetlow *et al*.^20^ showed that binding of enzymes to starch granules labeled by γ-^32P^-ATP *in vitro* resulted in enhanced amylase activity and increased amylose synthesis^19^,^20^,^21^. Recently, we used LC-MS/MS to identify several phosphorylation sites present in GBSS I and SS I^23^,^24^. Together with the development of phosphoproteomic approaches, TiO_2_ enrichment-based large-scale phosphoproteomic analysis has been performed in wheat and *Brachypodium distachyon*^25^,^26^,^27^. However, large-scale phosphoproteomic analysis of starch granule-binding proteins in cereal crops has not been performed due to two major limitations. First, it is difficult to enrich enough proteins from the starch granules for phosphoproteomic study because of the high sugar content and very low protein content of the granules, and the presence of numerous compounds that interfere with protein extraction. Second, knowledge of phosphorylation modifications in plant species, particularly in the very large genome of hexaploid wheat (up to 17 Gb), is limited.

In this study, we performed the first proteomic and phosphoproteomic analyses of starch granule-binding proteins, comparing the waxy wheat Shannong 119 and the bread wheat Nongda 5181. We also used dynamic transcriptional expression profiling and comparative analyses of the key genes related to starch biosynthesis during grain development to compare the two cultivars. Our results revealed the expression profiles of the key genes and enzymes, and their phosphorylated characterization *in vivo* involved in amylose and amylopectin synthesis, which provides new insights into the mechanisms of wheat grain starch biosynthesis.

## Results

### Dynamic Development of Grains and Starch Granules, and Starch Compositions of SN119 and ND5181

The growth period of waxy wheat SN119 was somewhat shorter than that of bread wheat ND5181. However, the grain size and weight of SN119 were significantly lower than those of the bread wheat ND5181. The thousand-kernel weight (TKW) of mature dry seeds in SN119 was 33.50 g, which was markedly lower than that in ND5181 (43.86 g; Supplemental Table S1). The starch in ND5181 was dyed red-brown, while that in the waxy wheat SN119 was stained blue (Supplemental Fig. S1A–C). However, the SEM observation of starch shapes during grain development in SN119 and ND5181 showed similar morphological features and developmental patterns. The A-granules were initiated at 10 DPA in both cultivars, while the B-granules emerged between 10 and 15 DPA (Supplemental Fig. S1D).

SDS-PAGE showed that the waxy protein that controlled amylose synthesis was absent in SN119 (Supplemental Fig. S2). Total starch and amylose contents five stages after flowering in SN119 and ND5181 are shown in Supplemental Table S2 and Supplemental Fig. S3. The total starch content increased from 10 DPA to maturity in both cultivars, and there were no significant differences between the cultivars. The total starch content in SN119 was 16.80% at 10 DPA, and increased to 63.64% at maturity. The starch content of ND5181 was 16.10% at 10 DPA and 65.05% at maturity. However, the amylose and amylopectin contents in SN119 and ND5181 were significantly different. As is shown in Supplemental Table S2, the content of amylose was increased from 2.43% at 10 DPA to 18.27% at maturity in ND5181, while the amylose content in SN119 was less than 3% at all developmental stages. In ND5181, amylopectin contents increased from 13.67% at 10 DPA to 48.21% at maturity, but in SN119 amylopectin contents increased from 16.27% at 10 DPA to 60.81% at maturity. However, the amount of amylopectin in single seeds was similar: 0.02037 g and 0.02047 g in SN119 and ND5181, respectively. The reduced amylose content had few effects on amylopectin synthesis, but led to significant reductions in total starch biosynthesis, grain size and weight, and grain yield.

### Comparative Proteome Analysis of SN119 and ND5181

Purity of the extracted starch granules was monitored using SEM and microscopic images, which showed no contamination with proteic or cell debris (Supplemental Fig. S4). The granule surface-associated proteins were removed by three washings with aqueous buffer containing 2.3% SDS and 1% DTT. SDS-PAGE analysis confirmed the complete removal of granule surface-associated proteins (Supplemental Fig. S5). Moreover, SDS extraction and sonication did not alter the appearance of starch granules, as observed using SEM.

**Figure 1:**
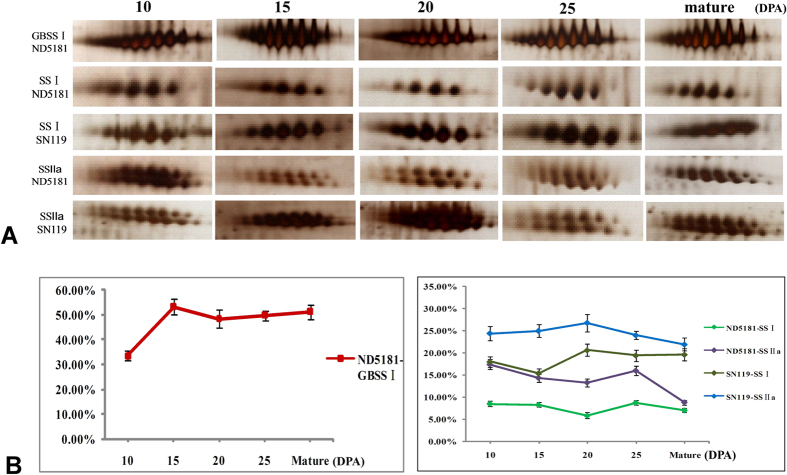
Comparison of the expression patterns of three starch granule-binding proteins in SN119 and ND5181. (**A**) Close-up views of expression levels for GBSS I, SS I and SS IIa protein spots on 2-DE gels. (**B**) Expression patterns and proportion of GBSS I, SS I and SS IIa proteins in five stages of seed development in SN119 and ND5181.

The dynamic expression patterns of SS I, SS IIa and GBSS I at five grain developmental stages in SN119 and ND5181, detected by 2-DE, are shown in Fig. 1 and Supplemental Fig. 6. Multiple isoforms were present in SS I, SS IIa and GBSS I (Fig. 1A). GBSS I had at least 10 isoforms in ND5181, and SS IIa and SS I had at least 12 and 5 isoforms in both SN119 and ND5181, respectively (Fig. 1A). The waxy protein content increased from 10 DPA to 15 DPA in ND5181, and then reduced slightly in later periods. SS I and SS IIa exhibited small changes during five stages in both varieties and showed a down-up-down pattern in ND5181, with the greatest quantity at 25 DPA. SS IIa displayed an up-down pattern, while SS I showed a down-up-down pattern in SN119, with the highest quantity at about 20 DPA (Fig. 1B).

**Figure 2:**
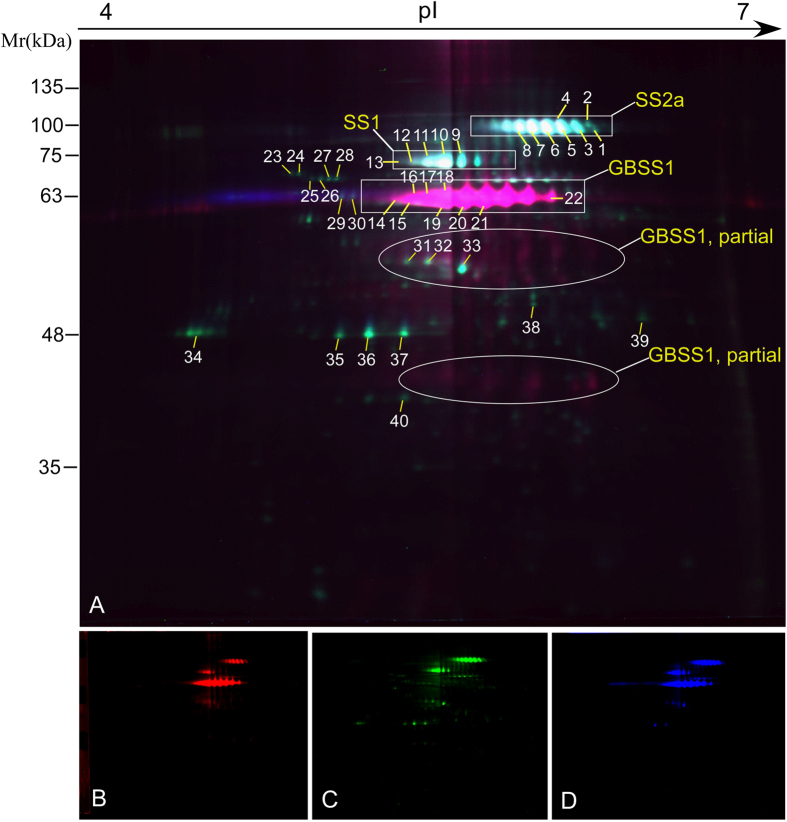
2D-DIGE images of proteins in ND5181 and SN119. (**A**) Labeled proteins were visualized for all fluorophores. (**B**) Cy2, red, mixture of equal amounts of all proteins as the internal standard. (**C**) Cy5, green, for the SN119 protein sample. (**D**) Cy3, blue, for the ND5181 protein sample.

Starch granule-binding proteins were extracted and the differentially expressed proteins (DEP) between SN119 and ND5181 were separated by 2D-DIGE (Fig. 2). In total, 40 DEP spots were recognized on the gels and identified by MALDI-TOF/TOF-MS. These comprised three types of protein: SS I, SS IIa and GBSS I/GBSS I partial (Supplemental Tables S3 and S4). There was no obvious difference in the quantity of SS I (spots 9–13) and SS IIa^1^,^2^,^3^,^4^,^5^,^6^,^7^,^8^ between SN119 and ND5181 (Fig. 2). For ND5181, the upregulated spots were mainly GBSS I (spots 14–22; Supplemental Tables S3 and S4).

### Phosphoproteome Characterization in Waxy and Non-waxy Wheat NCultivars

**Figure 3:**
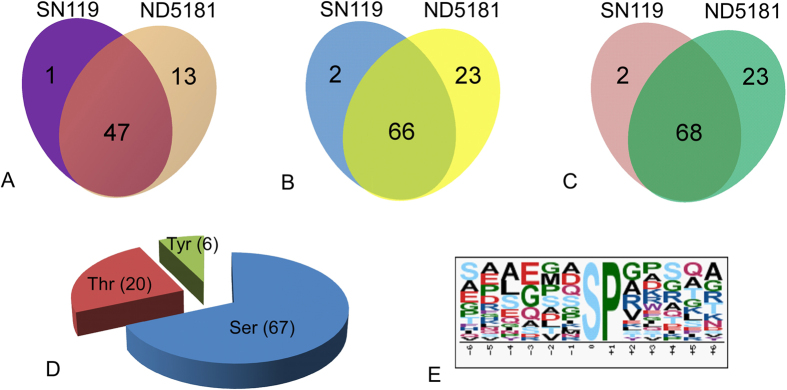
Phosphorylation analysis of SN119 and ND5181. (**A**) Phosphoprotein analysis of SN119 and ND5181. (**B**) Phosphorylated peptides analysis of SN119 and ND5181. (**C**) Phosphorylation sites of SN119 and ND5181. (**D**) Distribution of serine, threonine and tyrosine phosphorylation sites. (**E**) Analysis of the amino acids surrounding the identified phosphorylated residues by Motif-X.

The starch granule-binding proteins extracted from the purified starch granules at 20 DPA in both cultivars, with three biological replicates, were subjected to phosphoproteome analysis. The phosphopeptides were enriched using TiO_2_ microcolumns, and then identified by LC-MS/MS. A total of 93 phosphopeptides, containing 91 phosphorylated sites and 61 phosphoproteins, were identified (Fig. 3A–C). In ND5181, 89 phosphopeptides, containing 91 phosphorylated sites and 60 phosphoproteins, were identified (Supplemental Table S5). A total of 68 phosphopeptides, with 70 phosphorylated sites and 48 phosphoproteins, were identified in SN119 (Supplemental Table S5). Among the 93 phosphorylated sites, 67 Ser, 20 Thr and 6 Tyr sites were phosphorylated (Fig. 3D). Motif-X showed that only Ser motifs ([sP]) were enriched in both cultivars, based on accurate parameters (*P* < 10^−6^; Fig. 3E). Only phosphorylation sites P ≥ 0.75 were used for the subsequent analyses. All of the mass spectrometry phosphoproteome data obtained in this study are shown in Supplemental Table S4 and were deposited in the Proteome X change Consortium (http://proteomecentral.proteomexchange.org) via the PRIDE partner repository, with the dataset identifier PXD000646^28^.

The conserved phosphoproteins were mainly divided into the following categories: starch synthesis and branching enzymes, protein kinase/phosphatase, transporter-related proteins, transcription/translation factors, and others (Supplemental Fig. S7, Supplemental Table S6). Five starch synthesis enzymes (GBSS I, SS I, SS II-a, SBE I and SBE II-a) were phosphorylated and their phosphorylation sites were conserved in both SN119 and ND5181.

**Figure 4:**
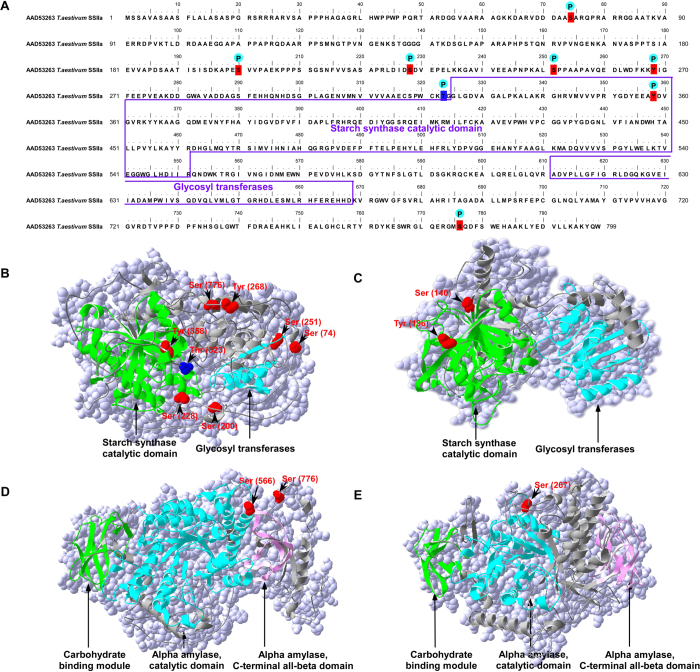
Phosphorylation of starch synthase-related enzymes. (**A**) Analysis of the amino acid sequence of SS IIa (phosphorylated residues are indicated). (**B–E**) Three-dimensional structures of SS IIa, SS I, SBE I, SBE IIa. The red balls represent the common phosphorylated proteins in both SN119 and ND5181, and the blue ball represents the special phosphorylated protein only existed in SN119.

We further analyzed the phosphorylation sites and 3-D structures of phosphoproteins SS I, SS II-a, SBE I and SBE II-a (Fig. 4). Eight phosphorylated sites were detected in SS II-a: five Ser (Ser74, Ser200, Ser228, Ser251, and Ser776), one threonine (Thr323), and two Tyr (Tyr268 and Tyr358; Fig. 4A,B) sites. Among them, seven phosphorylated sites (Ser74, Ser200, Ser 228, Ser251, Ser776, Tyr268 and Tyr358) were conserved in both cultivars. One Thr (Thr323) site was detected only in SN119 (Fig. 4B). Two conserved phosphorylated sites (Ser140, Tyr196) in SS I were detected in both ND5181 and SN119 (Fig. 4C), and two Ser (Ser566, Ser776) in SBE I and one serine (Ser267) in SBE IIa were found to be phosphorylated in both cultivars (Fig. 4D,E).

The phosphorylation levels of starch synthesis and branching enzymes were conserved, while those of other proteins were variable. The phosphorylation levels at 36 phosphorylated sites, corresponding to 19 phosphoproteins, were significantly changed (PLSC; Supplemental Fig. S8). Of the 36 phosphorylated sites, 14 were detected in SN119 and 22 phosphorylated sites were detected only in ND5181. Ser600 of glucose-6-phosphate isomerase was found to be phosphorylated only in ND5181, while the Tyr243 and Tyr249 sites of the vacuolar cation/proton exchanger were phosphorylated only in SN119 (Supplemental Fig. S8).

### Pro-Q Diamond Staining, Western blotting and Immunolocalization

**Figure 5:**
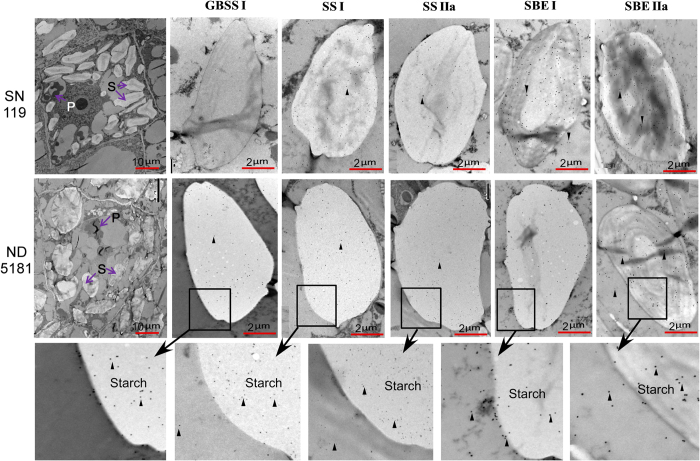
Immunolocalization of GBSSI, SS I, SS IIa, SBE I and SBE IIa in immature seeds (15 DPA). S, starch granules; PB, protein body; Triangular arrowheads indicate gold particles.

To further confirm the phosphorylation of the five main proteins (GBSS I, SS I, SS IIa, SBE I and SBE IIa) involved in starch biosynthesis, starch granule-binding proteins from 20 DPA in both cultivars were extracted, fractionated by SDS-PAGE, and silver-stained (Fig. 5A). The isolated proteins were identified by MALDI-TOF/TOF-MS. Western blotting showed that polyclonal antibodies against SBE I and SBE IIa, and monoclonal antibodies against GBSS I, SS I, and SS IIa demonstrated high specificity for SN119 and ND5181 (Supplemental Fig. S9). Furthermore, the starch granule-binding proteins at five developmental stages stained strongly with Pro-Q Diamond (Supplemental Fig. 10). GBSS I, SSI, SS IIa and SBE IIa were phosphorylated at all five stages, with similar phosphorylation levels in SN119 and ND5181. GBSS I and SS IIa were further detected using Phos-tag™, as described by Kinoshita *et al*.^29^ both proteins were phosphorylated (Supplemental Fig. S9C). Phosphorylation of SS I, SBE I and SBE IIa was not detected (Supplemental Fig. S9C), probably due to fewer phosphorylated sites and lower phosphorylation level.

To date, little is known about the localization of the main starch granule-binding proteins related to starch biosynthesis. In this study, we determined the subcellular localization of GBSS I, SS I, SS IIa, SBE I and SBE IIa in SN119 and ND5181. As shown in Fig. 5, the hybridization signal of GBSS I was present mainly in the starch granules of ND5181, while few signals appeared in waxy wheat and the cytoplasm. SS I and SS IIa were present mainly in the starch granules and few signals were detected in cytoplasm. SBE I and SBE IIa showed no specificity for starch granules or the cytoplasm.

### Dynamic Transcriptional Expression Profiling of Starch Synthesis-related Genes during Grain Development

**Figure 6:**
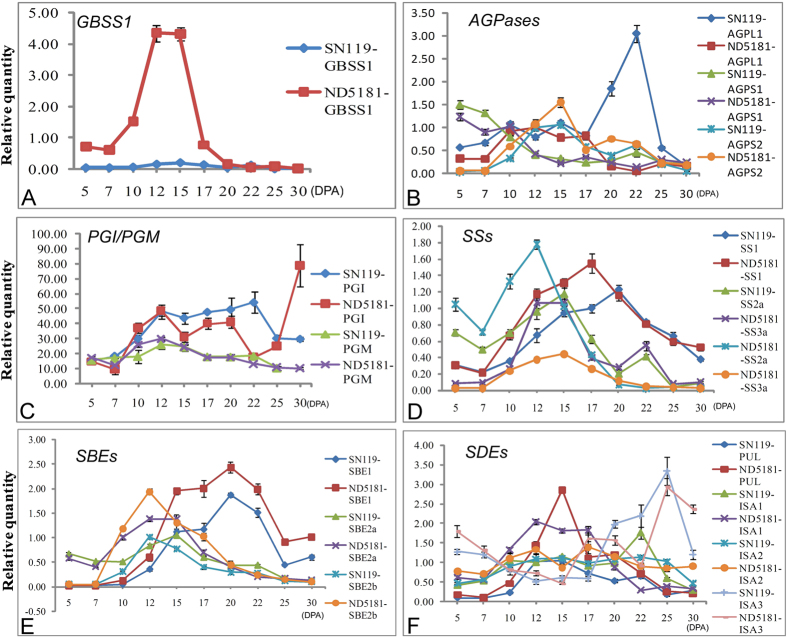
Quantitative real-time PCR (qRT-PCR) analysis of 6 key genes related to starch synthesis in developing seeds.

The dynamic expression profiles of 16 main starch synthesis-related genes during 10 grain developmental stages in the waxy wheat SN119 and non-waxy wheat ND5181 were analyzed by qRT-PCR (Fig. 6). In general, starch synthesis-related genes in SN119 and ND5181 displayed similar expression patterns during grain development. Most of them showed an up-down expression trend and had higher expression levels during the middle stages of grain development, but *ISA III* and *AGPS I* displayed down-up and down expression trends, respectively. *GBSS I* exhibited an up-down expression pattern in the bread wheat ND5181, but was barely expressed in the waxy wheat SN119 (Fig. 6A). Four genes (phosphoglucomutase (*PGM*), Phosphoglucoisomerase (*PGI*), ADPglucose pyrophosphorylase large subunits (*AGPL I*) and ADPglucose pyrophosphorylase small subunits (*AGPS II*) involved in the early stages of starch biosynthesis were expressed early and their expression peaked at about 12 DPA (Fig. 6B,C). Five genes (*SS IIa*, *SS III*, *SBE I*, *SBE IIa* and *SBE IIb*), which control the synthesis of longer chains (B1/B2-chains) were highly expressed at 12–15 DPA, while *SS I* and *SBE I*, which control the synthesis of shorter chains (A-chains), were expressed abundantly at 17–20 DPA (Fig. 6D,E). DBEs mainly showed an up-down expression pattern, with the exception of *ISA III* (Fig. 6F). Dynamic transcriptional expression profiling of starch biosynthesis-related genes showed similar transcriptional expression profiles in the two cultivars. The transcriptional and protein expression patterns of SS I, SS IIa and GBSS I exhibited up-down trends in both SN119 and ND5181.

Although the expression patterns of SD119 and ND5181 during grain development were generally similar, the expression levels of some genes showed greater differences. The expression levels of the majority of genes in ND5181 were slightly higher than in SN119. The expression levels of seven genes (*SS I*, *SS II-a*, *SBE I*, *SBE II-a*, *SBE II-b*, *ISA I* and *PUL*) were slightly higher in ND5181 than in SN119. Six genes (*ISA II*, *ISA III*, *PGM*, *PGI*, *AGPS I* and *AGPS II*) showed similar expression levels. However, *SS III* and *AGPL I* were expressed more highly in SN119 than in ND5181.

## Discussion

### Transcriptional and Translational Regulation of Starch Biosynthesis

Amylopectin is composed of multiple clusters and each cluster includes various chains. The chains can be divided into three categories according to their length and position: A-chains, B-chains, and C-chains. A-chains are linked to other chains at their reducing ends, whereas B-chains carry one or more chains belonging to a cluster. Only C-chains contain a reducing terminal in an amylopectin molecule^10^. A previous study indicated that the expression levels of starch synthesis-related genes are in accordance with starch-chain synthesis. Genes controlling synthesis of B-chains were expressed earlier than were those related to A-chains^23^. In this present study, similar results were found in common wheat ND5181 and waxy wheat SN119. Genes participating in the early stage of ADP-glucose synthesis, including *PGM*, *PGI*, *AGPL I*, *and AGPS II*, expressed earlier at the mRNA level. The genes controlling the synthesis of longer chains (B1/B2-chains), including SS IIa, SS III, SBE I, SBE IIa and SBE IIb, were then expressed. Genes (SS I and SBE I) controlling the synthesis of shorter chains (A-chains) were expressed abundantly last. Also, the studies of Cao *et al*.^24^ and Stamova *et al*.^30^ on transcriptomic analysis of starch biosynthesis in the developing grains of wheat cultivars CB037, Bobwhite and Hereward reported results similar to those presented herein. We propose that the expression patterns of starch synthesis-related genes in hexaploid wheat are constant at the transcriptional level, and the expression of starch synthesis-related genes is in accordance with starch-chain synthesis. Genes participating in the early stage of ADP-glucose synthesis were expressed early, followed by the genes controlling synthesis of longer chains (B-chains), and finally genes controlling the synthesis of shorter chains (A-chains).

The definitions of proteins designated as ‘granule-binding’ are arbitrary, and are generally based on the ability of a protein to be retained within the starch fraction following vigorous aqueous (buffers, SDS- and proteinase treatments) and non-aqueous (acetone or ethanol treatments) extraction and washing techniques described in several research articles^31^,^32^,^33^. In this study, investigation of the subcellular localization of GBSS I, SSs and SBEs using the immunolocation method, indicated that starch granule-binding proteins had different distributions. GBSS I protein was present mainly in the body of starch. Most of the SS I and SS IIa colloidal gold antibody signals were present in starch granules, and some signals were found in the cytoplasm. However, the distributions of starch branching enzymes did not exhibit specificity. SBE I and SBE IIb were present mainly in cytoplasm or were attached to the starch granule surface.

iTRAQ-based quantitative proteome analysis has been used in our laboratory to evaluate the differential expression of SS I, SS IIa, SBE I and SBE IIb using a total protein extraction method^34^. The results suggested that these proteins are present in the cytoplasm or attached to the surface of starch granules. However, waxy protein was not detected in this study, despite the presence of large quantities. This indicates that GBSS I is present in starch granules.

In this study, we performed the first proteome analysis of starch granule-binding proteins in wheat at five developmental stages. As SBE I and SBE IIb might be present mainly in the cytoplasm or attached to the surface of starch granules, as reported by Bancel *et al*., detection of SBEs by 2-DE is problematic^35^. Only GBSS I, SS I and SS IIa were used for comparative analysis in this study. The starch granule-binding proteins showed a different expression pattern at the protein level in the two wheat varieties, with which the transcriptional level findings were not always in agreement. The starch granule-binding protein expressed later at the protein level than at the transcription level. For example, at the transcriptional level, *GBSS I*, *SS I* and *SS IIa* were expressed strongly at 12–15 DPA, whereas, at the protein level, they were strongly expressed at about 20–25 DPA (Fig. 6). Moreover, *SS I* and *SS IIa* showed different expression patterns in SN119 and ND5181. *SS I* and *SS IIa* showed a down-up-down pattern in ND5181, and their expression was highest at 25 DPA. *SS IIa* displayed an up-down pattern and *SS I* displayed a down-up-down pattern in SN119, with the highest expression at about 20 DPA. Overall, the expression trends of starch synthesis-related genes were similar at the transcriptional level in SN119 and ND5181, but showed a different expression pattern at the protein level, mainly due to the lack of waxy protein.

### Effect of the Lack Waxy Protein on Amylopectin Synthesis

Starch is composed of amylose and amylopectin, and amylose is embedded in the lamellae of amylopectin^36^. Amylose synthesis is controlled by GBSS I, and amylopectins are synthesized via concerted reactions catalyzed by AGPase, SS, SBE, and DBE. Previous research showed that SS I, SS IIa, and SBE IIb can form protein-protein complexes in a phosphorylation-dependent manner^11^. However, there are few reports of protein-protein interactions among GBSS I and other proteins related to amylopectin synthesis. GBSS I may have a role in synthesis of long-chain amylopectin^37^, but it is unknown whether GBSS I affects the activity and expression of enzymes related to amylopectin.

Some reports have indicated that mutations of genes related to amylopectin synthesis, including *SS IIa*, *SS IIIa*, *SBE IIa* and *SBE IIb*, can increase amylose content^17^,^18^,^38^,^39^. For example, mutations of SS IIIa in rice (*Oryza sativa* L.) did not change the endosperm starch content, but increased the content of amylose^30^. Asai *et al*.^31^ reported that SS IIIa-deficient mutants exhibited increased GBSS I activity. In a waxy mutant, the wheat starch did not affect crystalline polymorphism, granule size, morphology, or gelatinization temperature^40^. Although total starch content was not altered significantly, mutants in rice, maize, barley, and wheat had either a reduced level of or completely lacked amylose in their endosperm starch^41^,^42^,^43^,^44^. In this study, SN119 contained 63.64% starch in ND5181 65.05%. The quality of amylopectin was similar in SN119 and ND5181, and each seed of SN119 and ND5181 contained 0.02037 g and 0.02047 g amylopectin, respectively. We also found similar results in other wheat varieties, including waxy wheat NM1, BH and common wheat YM50, SN8355 (data not shown). The 2D-DIGE results indicated that the SS I and SS IIa protein contents in the two wheat varieties were similar, indicating that the deletion of *GBSS1* may have little effect on the expression of amylopectin synthesis-related enzymes. Our results suggest that the biosynthesis pathways of wheat amylose and amylopectin are relatively independent.

### Phosphorylation modifications Play Critical Roles in Starch Biosynthesis

Large-scale phosphoproteomic analyses have been conducted on several plant species, including *Arabidopsis thaliana*^45^,^46^,^47^,^48^,^49^,^50^, rice^48^,^51^, *Medicago truncatula*^52^,^53^, soybean (*Glycine max* L.)^49^,^54^, canola (*Brassica napus*)^49^, maize^55^,^56^,^57^ *Brachypodium distachyon*^58^ and wheat^26^,^59^. However, further research on large-scale mining of the phosphorylation sites in starch biosynthetic enzymes has not been performed. A previous study demonstrated that phosphorylation could improve the activity of enzymes in starch, and increase the number of starch granules^19^. Research on phosphorylation of the key enzyme in starch synthesis has focused mainly on protein–protein interactions after phosphorylation. Tetlow *et al*.^19^,^20^ presented the potential phosphorylation-dependent protein-protein interactions within amyloplasts of wheat endosperm. In maize, Liu *et al*.^60^,^61^ also detected protein–protein interactions, with phosphorylation between starch biosynthetic enzymes in the amyloplasts.

In this study, we detected and analyzed the phosphorylation sites of starch granule-binding proteins in the common wheat ND5181 and the waxy wheat SN119; the data indicated that they had highly conserved phosphorylation sites. Our experimental results showed phosphorylation of starch granule-binding proteins at all developmental stages, indicating that phosphorylation modifications play critical roles in starch biosynthesis.

Large-scale phosphoproteomic studies involving plants have shown that most phosphoproteins possess only one or two phosphorylated sites^49^. In the present study, similar results were obtained, and 95% of the phosphoproteins identified in the two cultivars were found to possess fewer than three phosphorylated sites. However, almost all of the detected phosphoproteins related to starch synthesis had multiple phosphorylated sites. In particular, GBSS I and SS IIa had nine and eight phosphorylated sites, respectively, suggesting that conserved and multiple phosphorylated sites play important roles in starch biosynthesis. A previous study indicated that, in most phosphoproteins, the phosphorylation sites were localized at the N- or C-termini, and outside the domain regions^49^. We obtained a similar result from a phosphoproteomic analysis of the phosphorylation sites of phosphoproteins related to starch synthesis; i.e., the phosphorylation sites were present mainly outside the domain regions. For example, among the eight phosphorylated sites in SS II-a, only Tyr358 was located in the starch synthase catalytic domain. The other seven phosphorylated sites were outside the domain regions (Fig. 4). As proteins function through the conserved domain regions, phosphorylation may have an adverse impact on domain-associated functions. Therefore, phosphorylation of most phosphoproteins tends to occur outside the domain regions, and phosphorylation may contribute mainly to affinity among starch granule-binding proteins and enhance the protein–protein interactions.

In addition to starch synthesis-related enzymes, other proteins—including kinases/phosphatases, ubiquitin-related protein, transporter proteins, transcription factors, and ATPase—were found to be phosphorylated. Protein phosphorylation and dephosphorylation are reversible processes performed by protein kinases/phosphatases in eukaryotic cells. In this study, seven protein kinases and one phosphatase were identified and found to be phosphorylated. Exclude proteins related to phosphorylated modification, protein E3 ubiquitin-protein ligase, mainly controlling the ubiquitination pathway was also detected to be phosphorylated^62^. In this study, some sugar transporters, such as monosaccharide-sensing protein 2 (MSSP2), were found to be phosphorylated. MSSP2 may participate in the transport of ADP-glu from the cytoplasm to starch^63^. Previous research showed the presence of DNA in the amyloplast^64^. In this study, several factors related to transcription/translation, such as the elongation factor Tu and the translation initiation factor, were found to be phosphorylated and had conserved phosphorylation sites. In addition, we found that some globulins were phosphorylated. Debiton *et al*.^65^ used 2-DE and MALDI-TOF/TOF-MS to determine that globulin was present in starch granules. Globulins are likely to be α-amylase inhibitors that increase the nucleation rate of wheat starch by promoting the formation of hydrogen bonds between starch chains^66^,^67^.

### Putative pathways of Amylose and Amylopectin Biosynthesis

**Figure 7:**
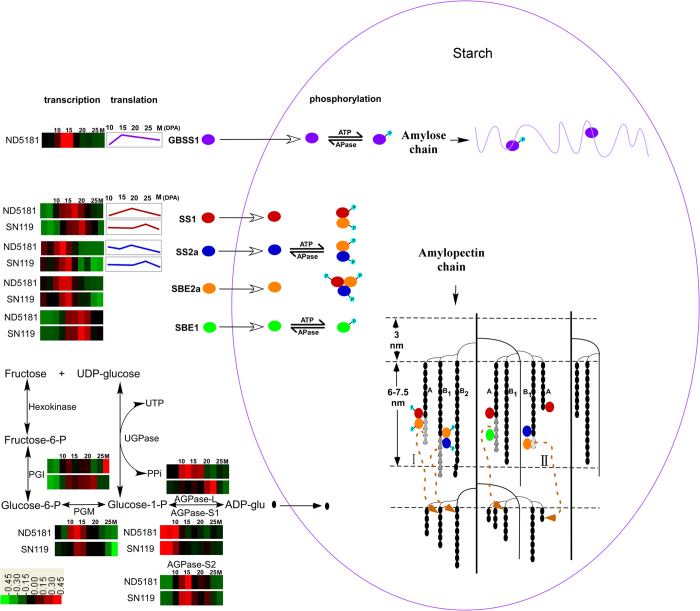
Schematic representation of amylose and amylopectin synthesis in the endosperm. The color coding of each row displays the changes in the expression of starch synthesis-related genes at the transcriptional level. The line graphs show the changes in the expression of of starch granule-binding proteins at the protein level. Phosphoproteins are indicated by lowercase letter p. Complexes consisting of SS I/SS IIa and SBE II were formed after phosphorylation, and these complexes synthesized and modified the chains. For example, following synthesis of excessively lengthy A/B1-chains by SS I/SS IIa, SBE II cuts and transfers them to form the next B1-chain.

We propose putative pathways of amylose and amylopectin biosynthesis, based on our results and those of previous reports. As shown in Fig. 7, ADP-glus are produced outside the starch by PGI, PGM, AGPase and other related enzymes. Starch synthesis-related genes function at transcriptional and translational levels in the cell nucleus and cytoplasm, respectively. The resulting enzymes—including GBSS I, SS I, SS IIa, SBE I and SBE II—are transported to starch, and are known as starch granule-binding proteins. The starch granule-binding proteins form protein complexes after phosphorylation. The protein complexes were synthesized and the chains are modified. For example, following synthesis of long A/B1-chains by SS I/SS IIa, SBE II cuts and transfers them to form the next B1-chain.

## Conclusion

In summary, we performed the first proteome and phosphoproteome analysis of starch granule-binding proteins in the waxy wheat SN119 and non-waxy wheat ND5181. 2D-DIGE revealed 40 DEP spots in waxy and non-waxy wheats, which belonged mainly to SS I, SS IIa and GBSS I. Most DEPs involved in amylopectin synthesis showed a similar expression pattern during grain development, suggesting the amylose and amylopectin synthesis pathways to be independent. A total of 91 phosphopeptides, containing 93 phosphorylated sites and representing 61 phosphoproteins, were detected in this study. The conserved phosphoproteins were divided into the following categories: starch synthesis-related enzymes, protein kinase/phosphatase, ubiquitin protein, transporter proteins, transcription/translation factors, and ATPase. Five starch synthesis enzymes (GBSS I, SS I, SS IIa, SBE I and SBE IIa) were found to have conserved phosphorylation sites, most of which were localized outside the domain regions. The expression patterns of the main starch synthesis-related genes of SN119 and ND5181 were similar at the transcriptional and translational levels. Our results revealed the expression features of the key genes and enzymes involved in amylose and amylopectin synthesis, and their *in vivo* phosphorylation, and provide new insights into the mechanisms of wheat grain starch biosynthesis.

## Materials and Methods

The strategy and experimental design of this study are presented in Supplemental Fig. S11. Developing grains from waxy and non-waxy wheat cultivars were harvested at 10, 15, 20, 25 and 30 days postanthesis (DPA), and the dynamic changes in grain morphology and starch granules were observed. Starch granules were separated and purified, and starch granule-binding proteins were extracted. The differentially expressed starch granule-binding proteins were separated by 2D-DIGE and identified by matrix-assisted laser desorption/ionization time-of-flight/time-of-flight mass spectrometry (MALDI-TOF/TOF-MS). Subcellular localization of key starch granule-binding proteins was determined using immunogold labeling. The dynamic transcriptional expression profile of key genes related to starch biosynthesis during grain development of the two cultivars were investigated. Starch granule-binding proteins extracted from 20 DPA grains, from both cultivars, were subjected to large-scale phosphoproteome analysis using TiO_2_ microcolumns and LC-MS/MS. The methods involved in this study are described in more detail below.

### Plant Materials, Planting, and Sampling

The bread wheat cultivar (2n = 6x = 42, AABBDD) Nongda 5181 (ND5181) and the waxy wheat cultivar Shannong 119 (SN119) were planted at the experimental station of the China Agricultural University (CAU), Beijing, during the 2012–2013 growing season (October–June). Nongda 5181 and Shannong 119 are newly developed cultivars by China Agricultural University and Shandong Agricultural University, respectively. Both cultivars have been released recently and began to application in the north China. Field experiments were performed in randomized block design with three biological replicates (each plot being of 36 m^2^). At the experimental location, the average annual amount of sunshine was 2690 h, and the average annual temperature was 2700 h, average annual temperature of 12.7 °C and approximately 150 mm rainfall occurred during the wheat growing season. Wheat plants were grown under identical natural soil conditions. Fertilizer (200 kg/ha urea, 400 kg/ha phosphate diamine [P_2_O_5_ 16%], 150 kg/ha K_2_SO_4_, and 15 kg/ha ZnSO_4_) was applied to soil prior to sowing. To facilitate harvesting grains at defined developmental stages, individual flowers were tagged using colored tape at various post-anthesis stages. Grains for qRT-PCR analysis were harvested at 5, 7, 10, 12, 15, 17, 20, 22, 25, 30 and 35 d post-anthesis (DPA) from the middle part of three areas. Grains from 10, 15, 20, and 25 DPA, and at maturity, were used for light microscopy and scanning electron microscopy (SEM) observation, and two-dimensional differential in-gel electrophoresis (2D-DIGE) analysis.

### Measurement of Total Starch, Amylose and Amylopectin Contents

Total starch and amylose contents were measured using total starch and amylose/amylopectin assay kits (Megazyme Int. Ireland, Ireland) by the concanavalin A (Con A) method, according to the manufacturer’s protocols.

### Light Microscopy and Scanning Electron Microscopy

A modified procedure of Guillon *et al*.^68^ was used for conventional chemical fixation. Grains were cut in transverse slices, approximately 3–4 mm thick, fixed in 3% (w/v) glutaraldehyde and 4% paraformaldehyde in 0.1 M PBS (pH 7.4) overnight, after which samples were directly dehydrated in a series of ethanol solutions in water and then infiltrated and polymerized in medium-grade LR white resin. For light microscopy analysis, sections approximately 1 μm thick were prepared, and I_2_/KI was used to stain the starchy endosperm.

Starch granules from different cultivars and grain developmental stages were dusted on the surface of a carbon-adhesive tab and sputter-coated with gold–palladium particles using Dentum Vacuum Desk II. SEM observation of starch granules was performed using a Hitachi Model S-4700 scanning electron microscope at 10.0 kV.

### Purification of Starch Granules

Starch granules at five grain developmental stages (10, 15, 20, and 25 DPA, and at maturity), and three biological replicates, were separated and purified according to Chen *et al*.^23^. Seeds were manually crushed after freezing in liquid N, and then soaked overnight in 500 mL of water at 4 °C. After centrifugation, 500 μL of water were added to the pellet. The slurry was wrapped in four layers of gauze to remove gluten components, and then centrifuged at 3500 g for 5 min. The supernatant was removed and the remaining pellet was collected. After centrifugation, 100 mL of deionized water were added to the pellet. The slurry was layered on 500 mL of 80% (w/v) CsCl, centrifuged at 3500 g for 5 min, and the supernatant was discarded. The precipitate containing the starch granules was then washed three times with 320 mL of washing buffer (55 mM Tris–HCl pH 6.8, 2.3% [w/v] SDS, 1% [w/v] dithiothreitol [DTT], 10% [v/v] glycerol) for 30 min at room temperature. At the beginning of each washing step, the granules were disrupted by sonication using an ultrasonic processor (Vibracell, VC50, Bioblock Scientific, Illkirch, France) at the power of 20 W with a 20 s pulse before continuous mixing. The starch granule pellet was also washed three times for 5 min with cold deionized water, once with cold acetone, and air-dried. Each washing step was followed by centrifugation at 3500 g for 5 min. All washing and centrifugation steps were performed at room temperature to avoid precipitation of SDS. Purity of the starch fraction was monitored using SEM. Microscopic images showed no proteic or cell debris contaminating the granules. Approximately 300 g samples of flour, representing every stage, were separated and purified.

### Extraction of Granule-Binding Proteins

The procedures of Bancel *et al*.^35^ and Cao *et al*.^24^ were used to purify wheat starch granules. Surface-associated proteins were removed by extensive washing in aqueous buffer containing 2.3% SDS and 1% DTT. SDS-PAGE was performed to test extracted proteins present in the supernatant at each washing step, and three washes were required to remove all contaminants. The effect of SDS extraction and sonication did not alter the visual appearance of starch granules, as observed by SEM. After removing the surface-associated proteins, starch granules (8 g) were first washed using 400 mL SDS buffer (62.5 mM Tris–HCl, pH 8.7, 2% [w/v] SDS, 10 mM DTT). The mix was sonicated at 200 W for 20 s, and then heated for 10 min at 100 °C under constant agitation. The slurry was cooled on ice for 5 min and then centrifuged at 16,000 g at 4 °C for 15 min. The pellet was subjected three times to the same washing step. Supernatant was collected after each washing and all supernatants were pooled. One volume of 30% (w/v) TCA in acetone was added to the supernatant for protein precipitation. The resulting pellet was washed twice with extremely cold (−20 °C) 80% (v/v) acetone and dried under a gentle stream of air. The dry pellet was suspended in 60 mL dense SDS buffer (0.1 M Tris–HCl buffer, pH 8.0, 30% [w/v] sucrose, 2% [w/v] SDS, and 5% [v/v] 2-mercaptoethanol) and 60 mL of Tris-saturated phenol (pH 8.0, Sigma). The mixture was gently shaken overnight at 4 °C and then centrifuged at 17,000 g for 5 min at 20 °C. The upper phenol phase was pipetted into a new tube, and this step was repeated twice. As a next step, five volumes of cold 0.1 M ammonium acetate in methanol were added to the phenol phase and the mixture was stored overnight at −20 °C. Precipitated proteins were recovered at 16,000 g for 5 min, and washed with cold methanolic ammonium acetate twice and cold 80% acetone twice. The final protein pellet was dried under natural conditions, and then dissolved in lysis buffer (7 M urea, 2 M thiourea, 4% w/v CHAPS and 65 mM DTT) overnight at 4 °C. The protein mixtures were harvested by centrifugation at 12,000 g for 15 min at 4 °C to remove insoluble material. The concentration of the protein mixture was determined with a 2-D Quant Kit (Amersham Bioscience) using BSA (2 mg/mL) as a standard. The final protein solution was stored at −80 °C for further use.

### 2D-DIGE, Image Acquisition, and Data Analysis

The modified procedure of 2D-DIGE was according to Gao *et al*.^69^. Protein samples were labeled separately as Cy2, Cy3 and Cy5. The sample labeled Cy2 was an internal standard prepared by pooling equal amounts of all samples. All samples were adjusted to pH 8.5 by adding 1 M Tris-base. Each 10 μL protein sample was labeled at the ratio of 400 pmol CyDye (GE Healthcare, USA) protein minimal labeling dye for 50 μg of protein on ice for 30 min, and then 1 μL of 10 mM lysine buffer was added to stop the reaction. The three labeled samples were combined into a single tube. Equal volumes of 2× sample buffer (7 M urea, 2 M thiourea, 2% w/v DTT, 4% CHAPS, 1% IPG buffer [pH 3–10], 0.004% bromophenol blue) was added to the labeled protein samples and left on ice for at least 10 min. Rehydration buffer (7 M urea, 2 M thiourea, 2% w/v CHAPS, trace bromophenol blue) containing 1% DTT and 0.5% IPG buffer (GE Healthcare) was added to bring volumes to 350 μL for IEF. The IEF and second dimension were according to Gao *et al*.^69^. To obtain adequate amounts of proteins from individual spots for protein identification, 50 μg of protein were extracted from each developmental stage and also run separately on conventional two-dimensional gel electrophoresis (2-DE) gels as described by Cao *et al*.^24^. The 2-DE experiments were repeated three times for error control.

The procedures to identify and analyze protein spots were according to Gao *et al*.^69^ and Cao *et al*.^24^. Image analysis was performed using Image Master 2D Platinum Software Version 7.0 (Amersham Biosciences). The experimental *M*r (kDa) of each protein was estimated by comparison with the *M*r markers, and the experimental *pI* as determined by its migration on the IPG strip. The protein spots were estimated by the percentage volume (% vol).

### Protein Identification Using MALDI-TOF/TOF-MS

Identification of the spots was performed by matrix-assisted laser desorption/ionization time-of-flight/time of-flight mass spectrometry (MALDI-TOF/TOF-MS). Protein spots were manually excised from the gels, and after trypsin digestion, they were analyzed on a 4800 Plus MALDI TOF/TOF Analyzer (Applied Biosystems, USA). All MS spectra were used for search in the National Center for Biotechnology (NCBI) database Viridiplantae (900091) and Triticum (16682) via the MASCOT software with GPS Explorer software version 2.0 (Applied Biosystems). The peptide tolerance was set as 100 ppm and fragment mass tolerance was 0.4 Da. One missed cleavage was allowed, and carbamidomethyl (Cys) and oxidation (Met) were specified as variable modifications. The results were creditable with Protein Score C.I.% and Total Ion Score C.I.% both above 95% and significance threshold p < 0.05 for the MS/MS^70^.

### Phosphopeptide Enrichment using TiO_2_ microcolumns

The procedure for enrichment of phosphopeptides from three biological replicates was as reported by Wu *et al*.^71^ and Zhang *et al*.^26^. Proteins extracted at 20 DPA were directly reduced with dithiothreitol (DTT), alkylated with iodoacetamide, and subsequently digested with endoproteinase Lys-C and trypsin. TiO_2_ beads (GL Sciences, Tokyo, Japan) were incubated in 400 μL loading buffer containing 65% acetonitrile (ACN)/2% trifluoroacetic acid (TFA)/saturated with glutamic acid. A total of 3 mg of tryptic peptides from every biological replicate was dissolved in 600 μL loading buffer, and then incubated with the appropriate amount of TiO_2_ beads. After washing with 600 μL buffer (65% CAN/0.1% TFA), the phosphopeptides were eluted twice with 300 μL elution buffer (500 mM NH_4_OH/60% ACN). The eluates were dried and reconstituted in 0.1% formic acid (FA)/H_2_O for MS analysis.

### Phosphopeptide Identification, Phosphorylation Site Localization and Bioinformatic Analysis

The raw files were processed using MaxQuant (version 1.2.2.5)^72^, and were then searched against the wheat database (77,037 entries). Up to two missing cleavage points were allowed. The precursor ion mass tolerance was 7 ppm, and the fragment ion mass tolerance was 0.5 Da for the MS/MS spectra. The false discovery rate (FDR) was set to <1.0% to identify both peptides and proteins. The minimum peptide length was set to 6. Phosphorylation residue localization was evaluated based on the PTM scores, which assign the probabilities for each of the possible residues according to their residue-determining ions. In this study, MaxQuant (version 1.2.2.5) was used to calculate the PTM scores and PTM localization probabilities. Potential phosphorylation residues were then grouped into three categories depending on their PTM localization probabilities; class I (localization probability, P ≥ 0.75), class II (0.75 > P ≥ 0.5), and class III (P < 0.5)^73^. A false discovery rate (FDR) of 1% was used to identify phosphorylation residues. Spectra without residue-determining ions led to the identification of phosphopeptides with undetermined residues. Phosphopeptides that met the following conditions were considered as having undergone a significant change in phosphorylation level according to the method described by Lv *et al*.^58^: (1) phosphopeptide detected in all three biological replicates, (2) phosphopeptides with P < 0.05 by Student’s *t*-test, (3) phosphorylation localization probability ≥0.75, and (4) phosphorylation site score difference ≥5.

The significantly enriched phosphorylation motif set was extracted from phosphopeptides with confidently identified phosphorylation sites (class I) using the motif-X algorithm (http://motif-x. med.harvard.edu/)^74^. The phosphopeptides were centered at the phosphorylated amino acid residues and aligned, and six positions upstream and downstream of the phosphorylation site were included. For C- and N-terminal peptides, the sequence was completed to 13 amino acids with the required number of X, where X represents any amino acid. Because the upload restriction of Motif-X is 10 MB, a FASTA format data set (nearly 10 MB) containing the protein sequences from the wheat protein database was used as the background database to normalize the scores against the random distributions of amino acids. The occurrence threshold was set to 5% of the input data, set at a minimum of 20 peptides, and the probability threshold was set to *p* < 10^−6^. The Phyre2 online server^75^ was used to predict the 3D structure of the proteins of interest. The 3D structures and the phosphorylated site were displayed using the SPDBV (version 4.1) software^76^.

### Antibody Development, Western Blot and Immunolabeling

Polyclonal antibodies were raised in rabbits against the synthetic peptides derived from the N-terminal sequences of *T. aestivum* SBE I, and *T. aestivum* SBE IIa^77^. Monoclonal antibodies anti-wheat GBSS I, SS I and SS II antisera were prepared as described by Li *et al*.^78^. The antigen was prepared by coupling the synthesized peptide to keyhole limpet hemocyanin using the heterobifunctional reagent m-maleimidobenzoyl-N-hydroxysuccinimide ester. The extracted proteins described above were separated by SDS-PAGE. After silver staining, the expected protein bands were collected and digested by trypsin, and then identified by MALDI-TOF/TOF-MS as described above. The identified proteins were further confirmed by Western blotting according to Li *et al*.^78^. Western blot analysis of phosphorylated protein was using Phos-tag™ as described by Kinoshita *et al*.^29^.

Immunolabeling experiments were performed with samples of 15-day-old immature grains, using methods described by Chen *et al*.^23^. The primary GBSS I, SBE I and SBE IIa antibodies were diluted 1:3000, and those against SS I and SS IIa were diluted 1:500. The samples were incubated with a secondary antibody. The goat anti-rabbit IgG antibodies for SBE I and SBE IIa, and goat anti-mouse IgG antibodies for GBSS I, SS I and SS IIa were conjugated to colloidal gold (Auroprobe-EM, GAR-G15; Janssen, Belgium) diluted 1:20 in blocking solution (5% skimmed milk, 0.1% Tween 20, and 0.2 M PBS) for 15 min. In the control experiment, the primary antibody was omitted to test for nonspecific secondary antibody binding^79^,^80^,^81^.

### RNA Extraction, cDNA Synthesis, and Quantitative Real-time Polymerase Chain Reaction (qRT-PCR)

Developing grains from the central part of the spikes in Nongda 5181 and Shannong 119 were harvested at 11 grain developmental stages (5, 7, 10, 12, 15, 17, 20, 22, 25, 30 and 35 DPA). Total RNA of individual samples was extracted with TRIzol reagent according to the manufacturer’s instructions, and mRNA was purified as described by Li *et al*.^42^ using oligo(dT) and random primers. cDNA was synthesized from approximately 100 ng mRNA using the Superscript first-strand synthesis kit (Promega, Madison, WI, USA). The resulting cDNA was used for qRT-PCR analysis.

Transcriptional expression patterns of these genes were determined using qRT-PCR according to Chen *et al*.^23^ with minor modifications. Primer pairs specific for the genes were designed using the Primer 5.0 software by imposing the following stringent criteria: melting temperature of 58 ± 2 °C, PCR amplicon length between 80 and 300 bp, primer length of 22 ± 4 bases, and 40–60% guanine-cytosine content. Primers were also designed within the 3′ region of each sequence to encompass all potential splice variants and to ensure equal RT efficiency. Each of the specific primers had a unique melting temperature peak. The efficiency of the primers was determined with standard curves generated using serial dilutions of the genes of interest. Efficiency ranged from 92 to 108%, and R^2^ values were >0.993 for expression analysis of the genes and the candidate reference genes (Supplemental Table S7). Triplicate PCRs were performed for each gene.

## Additional Information

**How to cite this article**: Chen, G.-X. *et al*. Biosynthesis and Regulation of Wheat Amylose and Amylopectin from Proteomic and Phosphoproteomic Characterization of Granule-binding Proteins. *Sci. Rep*. **6**, 33111; doi: 10.1038/srep33111 (2016).

## Supplementary Material

Supplementary Information

Supplementary Table S4

Supplementary Table S5
