# Supplementary material for: Biosynthesis and Regulation of Wheat Amylose and Amylopectin from Proteomic and Phosphoproteomic Characterization of Granule-binding Proteins
Source: Sci Rep. 2016 Sep 8;6:33111. doi: 10.1038/srep33111 (PMC5015113; doi:10.1038/srep33111)
Supplement: Supplementary Information [file srep33111-s1.pdf]

Biosynthesis and Regulation of Wheat Amylose and Amylopectin from Proteomic and Phosphoproteomic Characterization of Granule-binding Proteins

Guan-Xing Chen[1,3], Jian-Wen Zhou[1,3], Yan-Lin Liu[1,3], Xiao-Bing Lu[1],

Cai-Xia Han[1], Wen-Ying Zhang[2], Yan-Hao Xu[2], Yue-Ming Yan[1,2*]

Supplemental Fig. S1. Development and observation of seeds and starch granules in ND5181 and SN119. **A.** Whole seeds at the five stages of seed development in SN119 and ND5181. **B.** I_2-KI staining of starch storage in developing seeds at five stages. **C.** Iodine-stained bright-field images of sectional profiles for the visualization of starch. DPA: days post-anthesis. **D**. Scanning electron microscopy cross-sectional images of grains at various stages of development. The scale bar is 20 μm.

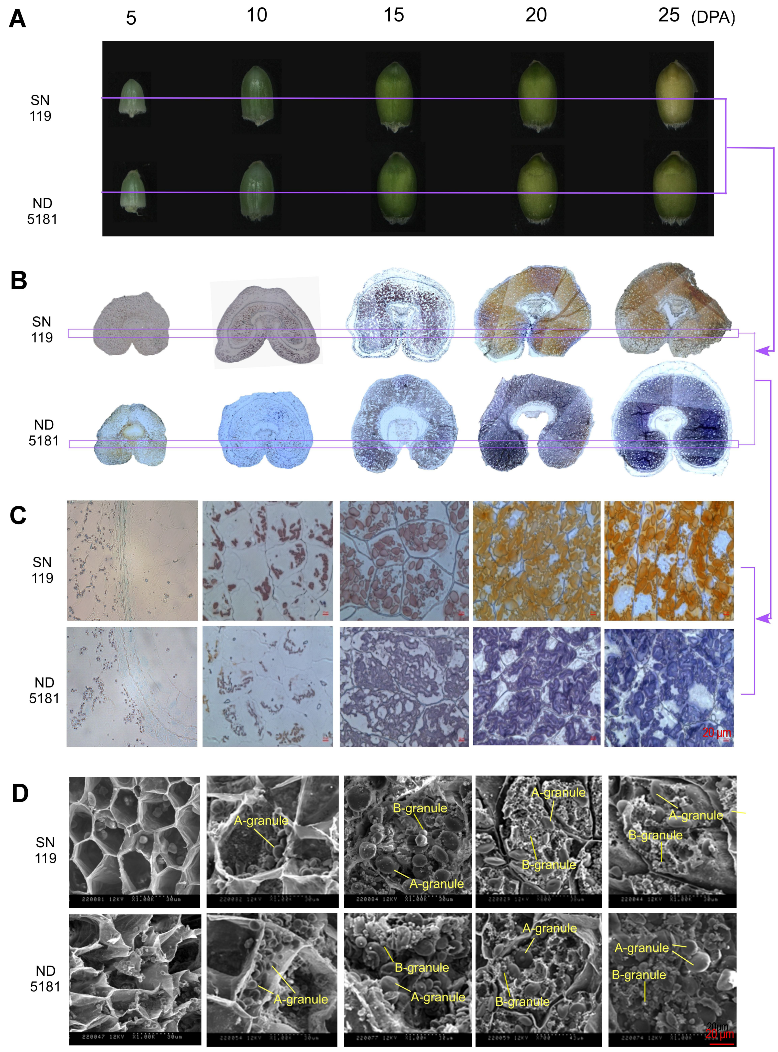

Supplemental Fig. S2. Silver-stained SDS-PAGE gel of starch granule-binding proteins in developing seeds of SN119 and ND5181.

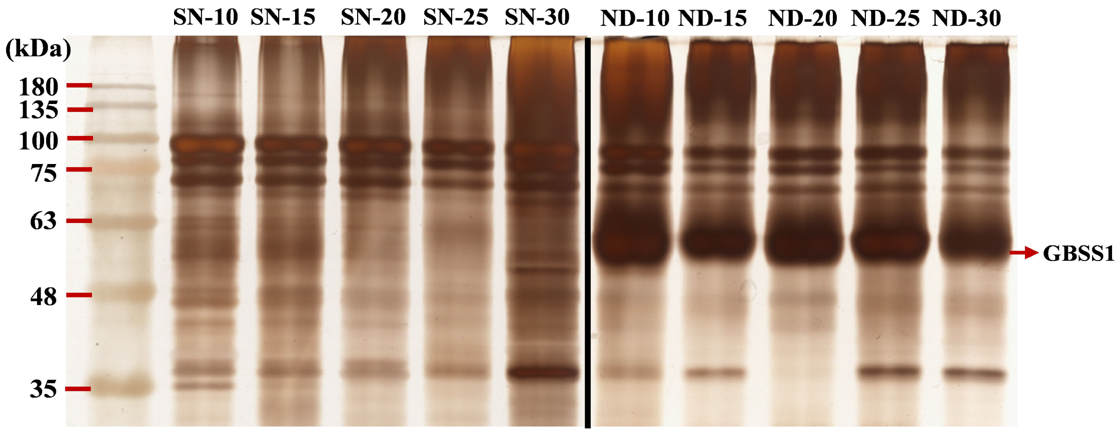

Supplemental Fig. S3. Comparison of starch, amylose and amylopectin contents in developing seeds of ND5181 and SN119.

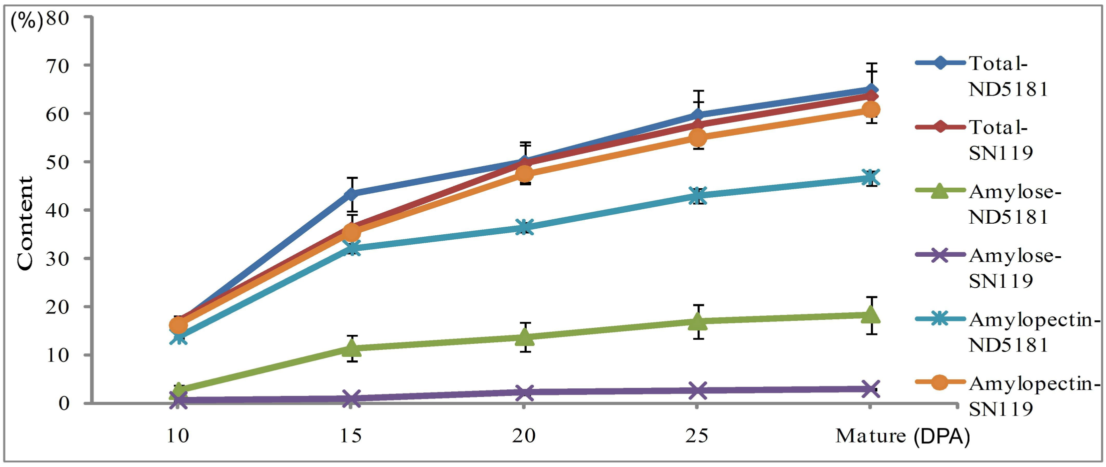

Supplemental Fig. S4. Scanning electron microscopy images for evaluation of the purity of the starch granules.

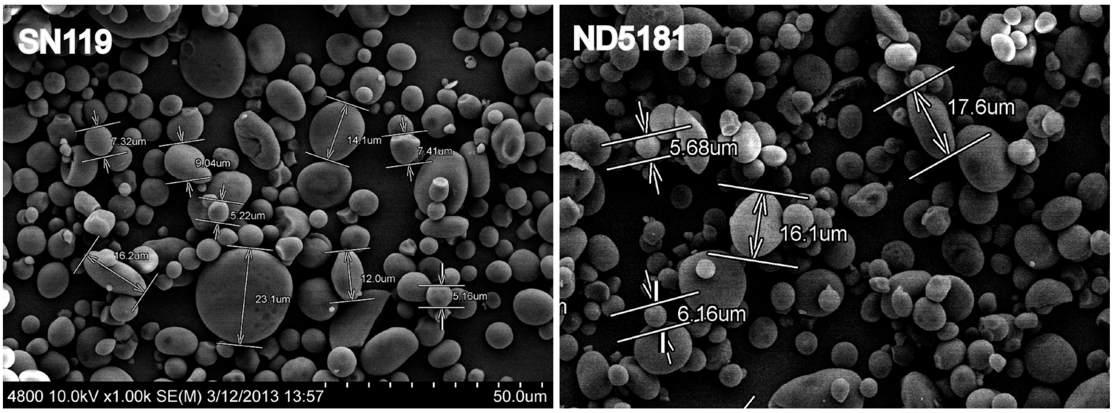

Supplemental Fig. S5. Silver-stained SDS-PAGE (T, 14%; C, 0.44%) patterns (1-6) of proteins in supernatant after successive washing steps (1-3).

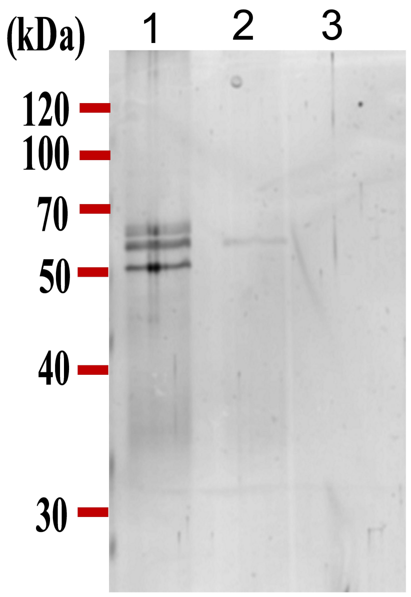

Supplemental Fig. S6. 2-DE gels of starch-associated granule proteins. **A.** The first row represents Silver-stained IPG (pH 4-7) × SDS-PAGE gels of starch-associated granule proteins in SN119. **B.** The second row represents Silver-stained IPG (pH 4-7) × SDS-PAGE gels of starch-associated granule proteins in ND5181.

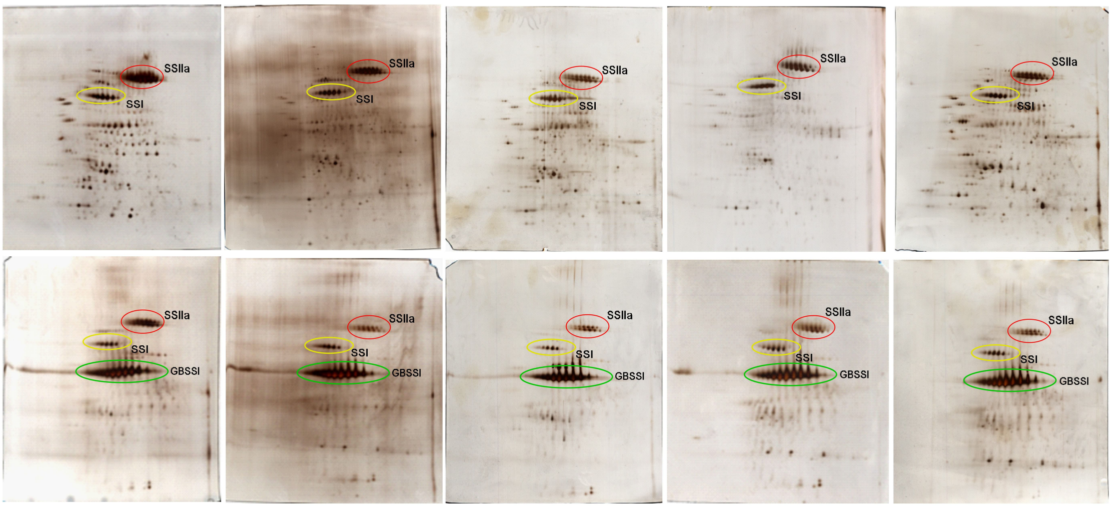

Supplemental Fig. S7. Molecular functions of phosphoproteins detected in starch of ND5181 and SN119.

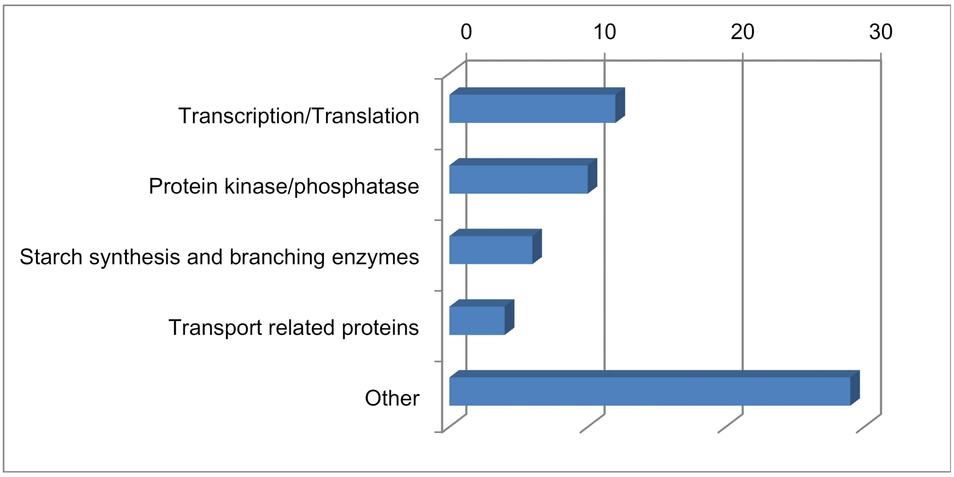

Supplemental Fig. S8. Analysis of significant changes in the phosphorylation level of phosphoproteins in SN119 and ND5181.

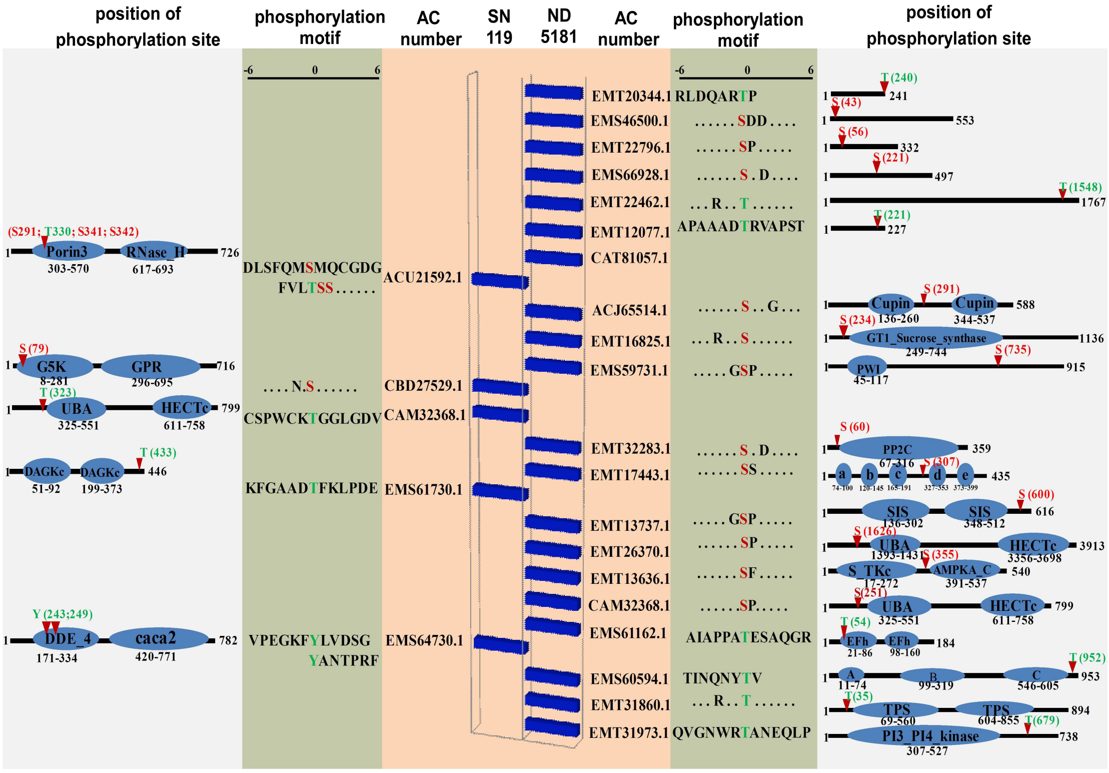

Supplemental Fig. S9. Starch granule-bound proteins from purified starch by electrophoretic analysis and Western blot. A. SDS-PAGE of amylase extracted from SN119 and ND5181. **B.** Detection and analysis of the antibodies of GBSS I, SS I, SS IIa, SBE I, and SBE IIa by western-blot. **C.** Phosphorylation of GBSS I, SS I, SS IIa, SBE I, and SBE IIa as determined by western blotting.

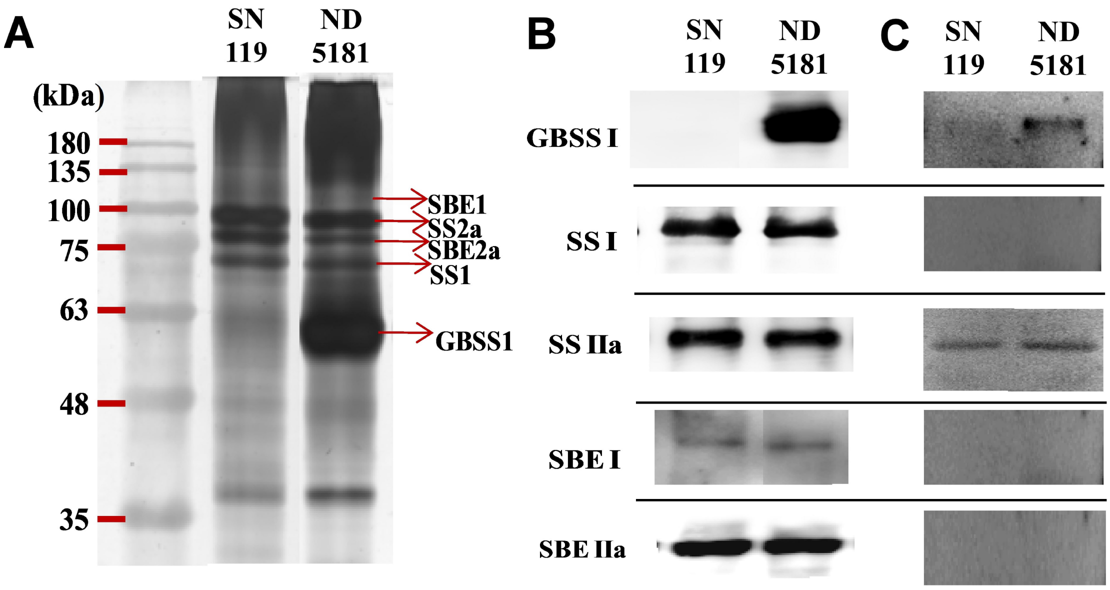

Supplemental Fig. S10. SDS-PAGE of starch granule-binding proteins revealed by Pro-Q Diamond in-gel staining. **A.** Silver-stained SDS-PAGE. **B.** Phosphoproteins detected by Pro-Q Diamond.

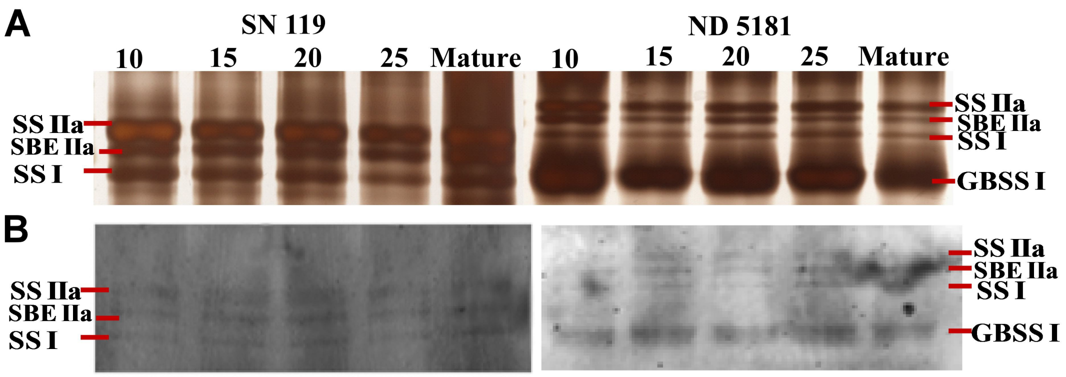

Supplemental Fig. S11. Experimental workflow adapted in the current study.

DPA: days post anthesis. 1, 2, and 3 represent the three biological replicates.

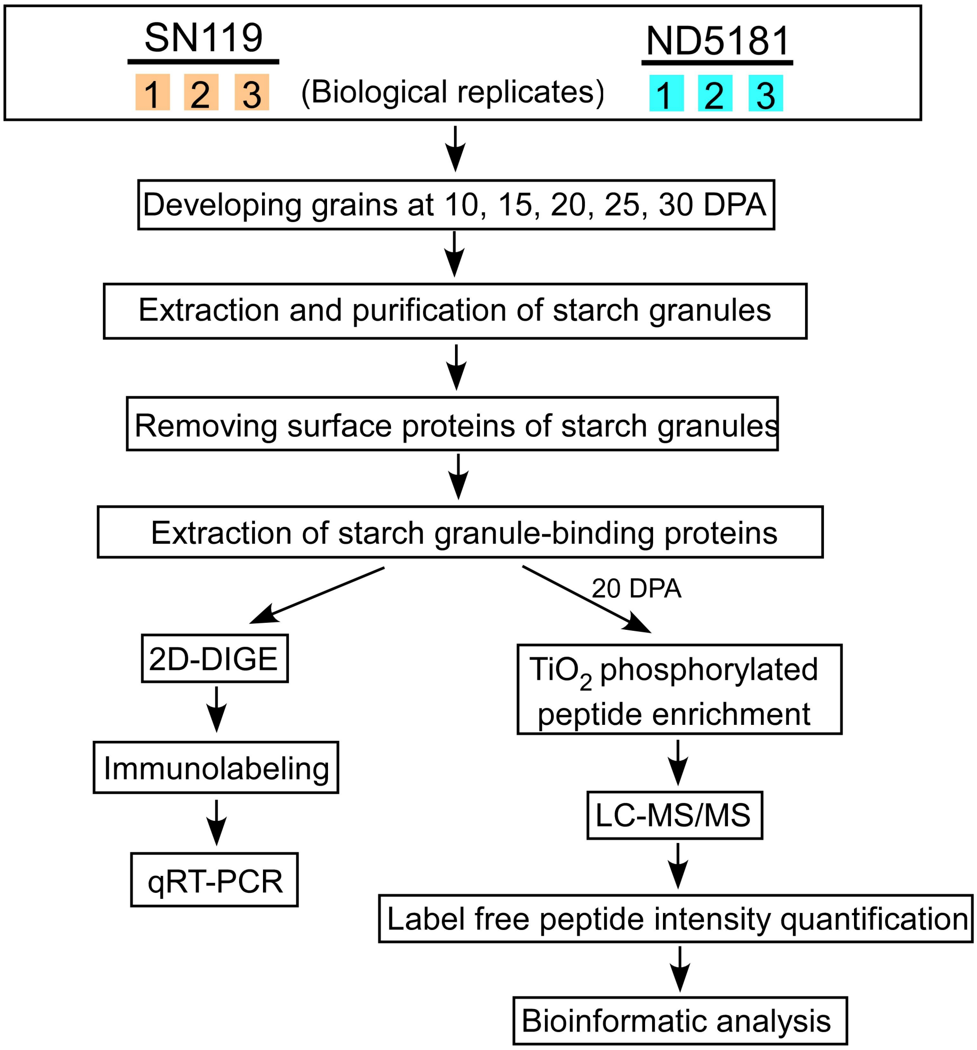

Supplemental Table S1. Comparison of yield traits between ND5181 and SN119.

Species	Total starch (%)	Amylose (%)	Amylopectin (%)	TKW (g)	Yield (Kg/hectare)
ND5181	65.05±1.48	16.84±0.98	48.21±2.34	43.9±6.32	8645**
SN119	63.64±1.31	2.83±0.11	50.81±2.12	33.5±4.23	5615

*P < 0.05, **P < 0.01

Supplemental Table S2. Comparative of starch content, amylose content and amylopectin content in development seeds of ND5181 and SN119.

Starch content (%)	ND-10	ND-15	ND-20	ND-25	ND-mature	SN-10	SN-15	SN-20	SN-25	SN-mature
Total starch	16.10	43.33	50.12	59.85	65.05	16.80	36.18	49.52	57.63	63.64
Amylose	2.43	11.35	13.63	16.84	18.27	0.53	0.85	2.03	2.57	2.83
Amylopectin	13.67	31.98	36.49	43.01	46.78	16.27	35.33	47.49	55.06	60.81

Supplemental Table S3. Granule-binding proteins identified by MALDI-TOF/TOF-MS in bread wheat ND5181 and waxy wheat SN119.

Spot	Protein Name	Accession No.	TMW/EMW	TP*I*/EP*I*	Coverage %	Number of peptides	Protein Score	Protein Score C. I. %	Total Ion C. I. %
1	starch synthase IIa-3	gi|8953573	87.1/107.0	6.27/5.76	44	32	781	100	100
2	starch synthase IIa-3	gi|8953573	87.1/124.0	6.27/5.80	27	17	231	100	100
3	starch synthase IIa-3	gi|8953573	87.1/110.0	6.27/5.83	30	19	433	100	100
4	starch synthase IIa-3	gi|8953573	87.1/124.0	6.27/5.68	31	19	266	100	100
5	starch synthase IIa-3	gi|8953573	87.1/108.0	6.27/5.70	50	34	864	100	100
6	starch synthase IIa-3	gi|8953573	87.1/109.0	6.27/5.64	47	23	700	100	100
7	starch synthase IIa-3	gi|8953573	87.1/108.0	6.27/5.60	35	25	753	100	100
8	starch synthase IIa-3	gi|8953573	87.1/109.0	6.27/5.56	34	25	300	100	100
9	granule bound starch synthase	gi|308229780	64.6/90.0	8.62/5.38	44	22	865	100	100
10	starch synthase I-1	gi|9369334	71.6/84.0	5.76/5.21	30	17	538	100	100
11	starch synthase I-1	gi|9369334	71.6/84.0	5.76/5.17	30	19	622	100	100
12	starch synthase I-1	gi|9369334	71.6/84.0	5.76/5.13	25	16	536	100	100
13	starch synthase I-1	gi|9369334	71.6/84.0	5.76/5.11	17	11	142	100	100
14	granule bound starch synthase	gi|308229780	64.6/62.5	8.62/5.12	41	21	898	100	100
15	granule bound starch synthase	gi|262385348	64.5/61.5	8.42/5.15	40	27	824	100	100
16	starch synthase (GBSSI)	gi|4760584	67.0/69.0	7.49/5.22	28	10	128	100	99.994
17	starch synthase (GBSSI)	gi|4760584	67.0/68.0	7.49/5.27	36	15	586	100	100
18	granule bound starch synthase	gi|308229780	64.6/68.0	8.26/5.32	38	17	299	100	100
19	granule bound starch synthase	gi|308229780	64.6/60.0	8.62/5.27	47	21	807	100	100

20	granule bound starch synthase	gi|308229780	64.6/60.0	8.62/5.37	40	16	627	100	100
21	granule bound starch synthase	gi|308229780	64.6/60.8	8.62/5.45	34	16	775	100	100
22	granule bound starch synthase	gi|308229780	64.6/64.0	8.62/5.67	47	20	448	100	100
23	waxy A1, partial	gi|334086818	64.1/38.5	8.73/5.00	32	16	354	100	100
24	granule bound starch synthase, partial	gi|378939984	64.3/47.0	8.61/5.87	42	19	556	100	100
25	granule-bound starch synthase precursor	gi|4588607	63.4/54.0	7.86/6.23	35	15	401	100	100
26	soluble starch synthase 1	gi|1373150	68/84.0	7.9/5.11	32	12	128	100	100
27	waxy A1, partial	gi|334086816	67.1/45.0	7.51/5.58	37	21	596	100	100
28	waxy A1, partial	gi|334086818	64.1/46.0	8.73/6.07	34	19	348	100	100
29	waxy D1, partial	gi|310619502	64.1/200.0	8.62/5.28	46	25	759	100	100
30	waxy D1, partial	gi|310619502	64.0/35.0	8.62/5.06	31	14	267	100	100
31	granule bound starch synthase, partial	gi|378939978	64.5/45.0	8.67/5.19	13	12	64	98.198	95.501
32	waxy D1, partial	gi|310619520	64.0/68.0	8.62/5.51	48	24	684	100	100
33	waxy D1, partial	gi|310619520	64.0/68.0	8.62/5.58	64	32	703	100	100
34	hypothetical protein CARUB_v10024064mg	gi|482563700	24.1/52.0	9.97/4.73	43	10	73	95.122	100
35	hypothetical protein	gi|473882607	19.7/47.1	4.65/4.71	19	33	75	99.87	100
36	retrotransposon protein, Ty3-gypsy subclass	gi|77553021	15.3/39.0	9.01/4.70	21	21	78	98.593	100
37	PREDICTED: outer membrane protein A-like	gi|449470698	37.5/30.0	5.19/5.71	27	9	242	100	100
38	PREDICTED: phosphoglycerate kinase-like	gi|449474076	41.4/35.0	5.1/5.93	18	5	92	99.939	100
39	PREDICTED: outer membrane protein A-like	gi|449470698	37.5/32.0	5.19/5.59	27	9	299	100	100
40	PREDICTED: outer membrane protein A-like	gi|449470698	37.5/32.0	5.19/4.86	27	9	165	100	100

Supplemental Table S6. Comparative analysis of phosphorylated sites identified in SN119 and ND5181.

AC number	Protein descriptions	Modified sequence	SN 119	ND 5181
		**Starch synthesis enzymes**		
P27736	Granule-bound starch synthase I	_FS(ph)FDDFAQLNLPDR_		+
		S(ph)PADAPLGMR		+
		LLKS(ph)VEEK		+
		LS(ph)VDCNVVEPADVK		+
		NCMIQDLS(ph)WK		+
		DAWDTS(ph)VVSEIK		+
		EEDVQIVLLGT(ph)GK		+
		KVVT(ph)TLK		+
		VVGT(ph)PAYHEMVK		+
		IYGPDAGT(ph)DYEDNQLR		+
CBN68294	starch synthase I	_S(ph)IVFVTGEAAPYAK_		
		ALY(ph)TAK		
CAM32368	starch synthase IIa	_T(ph)GGLGDVAGALPK_	+	
		Y(ph)IGFEEPVEAK		
		VDDDAAS(ph)AR		
		ALS(ph)PPAAPAVQEDLWDFK		+
		DAAEGGAPS(ph)PPAPR		
		LDIDS(ph)DVEPELKK		
		YGDYEEAY(ph)DVGVR		
AAG27621	starch branching enzyme 1	_DEGAAS(ph)WGK_		
		EMYTGMSDLQPAS(ph)PTIDR		
AAG27623	starch branching enzyme 2a	_IYESHIGM(ox)SS(ph)PEPK_		
EMS50245	1,4-alpha-glucan-branching enzyme	_LLISGAQGNPTS(ph)_		

Protein kinase/phosphatase

ABG68032	protein kinase	_MASWFETY(ph)HHPSR_		
EMS64674	Carbon catabolite-derepressing protein kinase	_S(ph)FNQFTSSESASPSTR_		+
EMT22775	Serine/threonine-protein kinase CTR1	CQTLIS(ph)GGVR_		
EMS49909	Bifunctional polynucleotide phosphatase/kinase	_DQTAVSPLEVPS(ph)PK_		
EMT27965	Cysteine-rich receptor-like protein kinase 27	_LFDSPQTY(ph)MT(ph)QSQDIK_		
EMS60500	Pantothenate kinase 2	_GGVDS(ph)PSWR_		
EMT28782	delta1-pyrroline-5-carboxylate synthetase	_LVNSS(ph)FADLQK_	+	
EMT31973	Phosphatidylinositol 4-kinase type 2-beta	_T(ph)ANEQLPTSASFVK_		+
EMT22829	phosphate dikinase 1	_GGMT(ph)SHAAVVAR_		
EMS64046	protein phosphatase 2C	_S(ph)ISAEGLHSLR_		+

Transport related proteins

CAY38593	ammonium transporter	_ISAEDEM(ox)AGM(ox)DLT(ph)R_		
EMS52034	Protein brittle-1, chloroplastic/amyloplastic	_LVS(ph)GAIAGAVSR_		
EMS57842	ABC transporter B family member 19	_DLGAAST(ph)R_		
EMS63940	Monosaccharide-sensing protein 2(carbohydrate transport)	_GGGQSALGSALGLMS(ph)R_		

Transcription/Translation

P41378	Eukaryotic initiation factor 4A	_VHACVGGT(ph)SVR_		
EMS49802	Nuclease domain-containing protein 1	_LWQYGDVES(ph)DEEDQAPGGR_		
EMS50100	Translational activator GCN1	_AILEGGS(ph)DDEGASTEAQGR_		
EMS66221	Pre-mRNA-splicing factor SYF1	_LEAAEEDGAAGS(ph)DEGEK_		
EMS67130	Splicing factor 3B subunit 4	_LLAANNPGS(ph)QK_		
EMS62939	Serine/arginine-rich splicing factor 7	_IGSGGLGS(ph)GR_		
EMT30106	Eukaryotic translation initiation factor 3 subunit G	_FGDAAS(ph)GDDASAR_		
EMT22662	SNF1-related protein kinase regulatory subunit gamma 1	_LGS(ph)FTFR_		
EMS55348	Elongation factor Tu, mitochondrial	_T(ph)VGAGVVAK_		

EMT33717	Far upstream element-binding protein 3	_SVIAEAEAGGS(ph)PALIAK_	
ACU21592	Zinc finger protein	_LQS(ph)PGAQQYYGTSR_	
		S(ph)SSFPAPQYALQR	+
		LLESPS(ph)GSAYAS	
		QGEASAGNQGMQS(ph)PYR	
EMS62713	Zinc finger CCHC domain-containing protein 8	_PSSGAFIDQSPT(ph)R_	
		DSSANQPAS(ph)PGTTFGNAGQLSK	
**Others**			
EMT31860	Alpha,alpha-trehalose-phosphate synthase (UDP-forming) 6	_VMT(ph)ASGIVPGLDR_	+
EMS60594	ATPase	_GLDIDTINQNYT(ph)V_	+
CAA72273	serpin	_AAEVTTQVNS(ph)WVEK_	
ACJ65514	globulin 3	_LGSLLGS(ph)R_	+
EMT13737	Glucose-6-phosphate isomerase	_ALIAEGSCGS(ph)PR_	+
EMT16825	Sucrose-phosphate synthase	_NFS(ph)DLSVWSDENK_	+
		SDDATEVSETDS(ph)PGDSLR	
EMS58635	Mannose-1-phosphate guanyltransferase	_VSS(ph)FEALQSATK_	
CAA07610	phospoenolpyruvate carboxylase	_LSS(ph)IDAQLR_	
EMT10773	Glucan endo-1,3-beta-glucosidase GV	_CT(ph)AAMLR_	
EMS56599	2-isopropylmalate synthase A	_MVQEY(ph)S(ph)GLHVQPHK_	
EMS53525	serine incorporator	_AGSSATVLSAPDS(ph)PR_	
		VDGQAHTNEVS(ph)K	
		FQDQGGIMT(ph)AETGTYR	
		NTLVS(ph)GGVR	
		CFAGKPS(ph)EQEK	
		VQSGVM(ox)T(ph)AETGTYR	
EMS59731	Serine/arginine repetitive matrix protein 1	_VDEASQSDGGS(ph)PLQK_	+

ACV44213	WALI7	_INS(ph)MPR_	
		QVAHAPQELNS(ph)PR	
		VDS(ph)EGVMCGANFK	
EMT26370	E3 ubiquitin-protein ligase UPL1	_DNS(ph)PTQTSVVIDDSK_	+
ACN54191	ubiquitin-like protein	GGAGDGEGAGS(ph)ESPPSGAR_	
EMS60602	BEACH domain-containing protein lvsC	_KIS(ph)NSENQLVK_	
EMS65816	Exocyst complex component 2	_LITEASMSS(ph)PVSR_	
EMT13238	Acidic leucine-rich nuclear phosphoprotein 32-related protein 1	_AVEAALHTAGEGSSS(ph)PAR_	
EMT32010	Calmodulin-binding transcription activator 3	_VQS(ph)FQR_	
EMS61162	calcium-binding protein CML16	_AIAPPAT(ph)ESAQGR_	+
		EEELS(ph)PVAEAAPVTAR_	
EMT14157	Sorting and assembly machinery component 50-A-like protein	_LVPGESEDGS(ph)PAVPGR_	
EMS63456	COP9 signalosome complex subunit 6a	_AQASFPAEAPS(ph)PGK_	
EMS64992	Chaperone protein dnaJ 13	_MDS(ph)LSTQLTEAR_	
EMT25410	Hypothetical protein	_DDVGLPLT(ph)PK_	
EMT31601	hypothetical protein	_FADDIS(ph)PR_	
EMT25443	hypothetical protein	_S(ph)PTPQQTGNR_	
EMT26919	hypothetical protein	_VEASGAYS(ph)NK_	
EMS55518	hypothetical protein	_S(ph)PVDGTTGTVR_	
EMT25637	hypothetical protein	_DVSSLGSAS(ph)PLQEQNQK_	

Supplemental Table S7. Efficiency and R2 values (coefficient of determination) of primer pairs. Data was calculated using standard curves

Gene	Forward primer(5'-3')	Reverse primer(5'-3')	Amplicon length (bp)	Efficiencies	R^2
ADP	GCTCTCCAACAACATTGCCAAC	GCTTCTGCCTGTCACATACGC	126 bp	100.7%	0.999
Ubi4	TGACACCATCGACAACGTGA	GAGGGTGGACTCCTTCTGGA	126bp	97.6%	0.983
GBSS Ⅰ	CTGGTCACGTCCCAGCTC	ACGAACACGAGGTTCATGC	321bp	96.6%	1.000
SS Ⅰ	GCAAAAGGAGAGGAGGGTACA	ACGTATGGTCTTTCGTCATGC	236bp	101.2%	0.999
SSⅡa	CCTGAGCACTACCTGGAACACTT	TCTGCCGTATGATGTCGTGAA	175bp	101.1%	0.999
SSⅢa	AGTCGGAGGGTTGGTTTA	ATCCCATTCCTGTTGTCATA	196bp	104.6%	0.996
SBE1	GGCCCAGTACAACGATCACT	CTTTGACACGAGTGGCTTCA	257bp	105.7%	0.997
SBE2a	TCGTGGCATAGCATTACAT	GAGTTTGCGGACCTCTTG	239bp	97.3%	0.999
SBE2b	CTTTGGTGGATTTGGTAGG	TTAGTTCATTGGAGCATAGACA	130bp	100.1%	0.996
ISA1	TGAATCGGACTGGGAACG	CACCACGACATTGGAAACAT	131bp	99.2%	0.993
ISA2	AGAGGCTAAGGCAGATAAGG	CGAACCAGCAGAGGAGTG	291bp	99.4%	0.999
ISA3	TGCGGGAATACACTAAACT	GAGGAGCATCAAGAGGACTA	157bp	106.9%	0.985
PUL	TCATCTCCGCTTTTTCGTCT	TACTTCGTGCGGACACACAT	272bp	104.2%	0.999
AGPL1	GTCACATTCACCGCACCT	GTCCTCAAGCACCCAGATA	152bp	102.2	0.999
AGPS1	CTTCTATGACCGTTCTGCC	CAACCTTCACCGATAACAC	102bp	109.5	0.999
AGPS2	CGATGGTTACTGGGAAGA	TTTGAAGGAGGCAAGTGT	144bp	104.7	1.000
PGI	AATCCATCAGGGAAGAGTT	ATAAGCAAGTGCGTCAGG	142bp	103.5	0.997
PGM	TGGCTAACCTAGTCAAGATG	CTGCTCAATGTAAATACGG	239bp	106.4	0.998

Primer pairs efficiencies and R2 values (coefficient of determination) calculated for standard curves (5-fold dilution series from pooled cDNAs) in the species of CS and *Ae. peregrina*.
